# Dietary Determinants of Metabolic Syndrome Parameters Differ by Gender in College Students

**DOI:** 10.3390/nu11122892

**Published:** 2019-11-27

**Authors:** Sara Saltzgiver, Alexander Nielson, Heidi Costello, Adam Baker, Julian Chan, David Aguilar

**Affiliations:** 1 Department of Exercise and Nutrition Sciences, Weber State University, Ogden, UT 84408-2801, USA; sarasaltzgiver@mail.weber.edu (S.S.); Heidicostello@weber.edu (H.C.); Adambaker1@mail.weber.edu (A.B.); 2 Department of Mathematics, Weber State University, Ogden, UT 84408-2801, USA; Alexandernielson@mail.weber.edu (A.N.); Julianchan@weber.edu (J.C.)

**Keywords:** decision tree, HDL-C, blood pressure, MyPlate, food groups, MetS

## Abstract

MyPlate is a guidance system for healthier eating choices. In this cross-sectional study, we investigated the influence of MyPlate food group consumption and exercise on metabolic syndrome (MetS) parameters in college students. Participant (*n* = 462) blood was analyzed using Cholestech for triglycerides (TG), glucose, low-density lipoprotein cholesterol (LDL-C), and high-density lipoprotein cholesterol (HDL-C). In addition, weight, waist circumference (WC), and blood pressure (BP) were measured. Diet and Wellness Plus was used to compute participant diet records. Regression analysis and a recursive decision tree were made to predict MetS using RStudio (V.1.1.463). BP decision tree predicted high risk of elevated blood pressure with a recall rate of 93.7%. For males; exercise, empty calories, dairy, and protein were main predictors. For females, vegetable and empty calorie consumption were primary determinants. HDL-C decision tree had a recall rate of 91.8% and showed that the main low HDL-C risk determinants for males were; exercise and grain consumption. Conversely, for females; empty calories, grain, and vegetable consumption were the key factors determining low HDL-C risk. This study shows that MyPlate recommendations are valuable to achieve adequate HDL-C and blood pressure and provides insight into the importance of tailoring food intake guidance based on gender.

## 1. Introduction

Metabolic syndrome (MetS) is a co-occurrence of factors that are associated with increased risk for chronic disease. According to the National Cholesterol Education Program Adult Treatment Panel III (NCEP ATP III) criteria the presence of three or more of the following parameters is indicative of MetS: high waist circumference, elevated serum triglycerides, reduced high-density lipoprotein cholesterol, elevated fasting blood glucose, and hypertension [1]. The measurement specifications of each parameter are included in Table 1.

It is estimated by the National Health and Nutrition Examination Survey (NHANES), that in 2006, approximately 25.0% of the United States adult population had MetS [2]. Then, from 2007 to 2012, the incidence of MetS dramatically increased to 34.2% [2]. Affecting more Americans than ever before, MetS has quickly become recognized as a major precursor to potentially chronic health complications and disease in this country.

Short term complications of MetS can include atherosclerosis, insulin resistance, kidney disease, etc. [3]. The prolonged prevalence of MetS parameters can lead to more serious chronic diseases such as cardiovascular disease, stroke, and type 2 diabetes [4]. Cardiovascular diseases are responsible for over 610,000 deaths each year in the United States [5]. Over 140,000 lives are claimed from stroke, while nearly 750,000 people suffer from a stroke each year [6]. The CDC also reports that 30.3 million individuals in the U.S. are type 2 diabetic, and more than 84.1 million are prediabetic [7].

MyPlate is an online dietary recommendation platform to promote healthy eating and reduce the risk of diet-related diseases. It is published by the USDA Center for Nutrition Policy and Promotion and is a widely used tool by registered dietitian nutritionist to provide nutrition advice [8]. This tool serves the public with a simple method for understanding the current dietary guidelines for Americans. The MyPlate system divides food into five distinct food groups: fruits, vegetables, grains, protein foods, and dairy products [9]. In addition to food groups, MyPlate also emphasizes moderation in empty calories which are calorically dense food items that lack essential micronutrients and dietary fiber [10]. MyPlate recommendations for both empty calories and food group consumption are based on factors including height, weight, age, gender, and physical activity [9]. Even with these somewhat individualized and simplified dietary calculations, many limitations still prevent individuals from achieving MyPlate goals [11]. Understanding these limitations could be key to facilitating the implementation of this dietary patterning model to a broader audience. Personal preferences towards and against specific food groups and/or empty calorie food items play a big role in whether individuals will follow MyPlate recommendations.

Previous studies have shown that men and women have distinctly different food preferences. In general, women tend to prefer fiber-rich food groups (vegetables and fruit), and high-calorie food items more than men do, while men are more inclined to consume high fat and high protein food groups [12,13,14]. Physiological, behavioral, and/or social differences among the sexes may be responsible for part of the differences in food group preferences [15]. These factors may include differences in hormone concentration, neuroendocrine signaling, pregnancy/lactation, fungiform papillae density, olfactory reception, body composition, caloric expenditure, caloric need, societal pressures that encourage dieting, desire for weight control, and/or perceived health benefits of certain food groups [16,17,18,19,20,21].

The main purpose of this study was to identify food group(s) that are most determinant in the development of metabolic syndrome that are specific for gender. Furthermore, we aimed to create a model that predicts the statistical likeliness of metabolic syndrome based on such factors, establishing a framework to promote more gender-specific recommendations.

## 2. Materials and Methods

### 2.1. Data Collection and Analysis Methods

This cross-sectional study (IRB# 16-ED-062) includes tests and records gathered from Weber State University students, collected between 2015 and 2018. Participants were part of an introductory nutrition course and volunteered to take part in the study. Among the 462 subjects, the study included 153 men and 309 women. Sample age ranged from 18 to 65 years, with the average age being 24 years. A two-day diet record was submitted by each participant. Subjects also participated in anthropometric and blood testing to gather measurements for MetS parameters. Parameter measurements for height, weight, waist circumference (WC), BMI, blood pressure, blood glucose, serum triglycerides, and HDL-cholesterol were collected and used for data analysis. All tests were collected in Weber State’s Human Biochemistry Laboratory.

### 2.2. Anthropometrics

To the nearest centimeter, height was measured with a standardized stadiometer while the participant was facing straight forward, with good posture, and no shoes on. Weight was recorded at the nearest 0.1 kg, using a weight beam scale. Both height and weight measurements were used in the Quetelet index equation to calculate body mass index (kg/m^2^). Blood pressure was measured using an automatic digital sphygmomanometer on the left arm at the beginning of the examination, and again after 5–10 min of seated rest to ensure accurate results. Both systolic and diastolic BP were recorded in standard units of mmHg.

Waist circumference was measured to the nearest 0.1 cm, using a flexible, non-stretch measuring tape. Participants were instructed to stand with feet together, arms at their sides, and with normal posture. Circumference measurements were made directly superior to the iliac crest of the right and left os coxa bones. This measurement was tested three times and recorded as a mean average value.

### 2.3. Diet Records

Participants were instructed to log their normal food and water intake over a two-day period. Diet records were tracked using Diet and Wellness Plus Cengage software. The following reports were computed and submitted in each diet record: “Intake vs. Goals”, “Macronutrient Ranges”, “Fat Breakdown”, “MyPlate Analysis”, “Energy Balance”, “Daily Food Log”, and “Daily Activity Log”.

This study specifically examined the “MyPlate Analysis”, which lists governmental goals for food groups, in comparison to the actual intake per day of each food group for grains, vegetables, fruits, dairy, protein foods, and empty calorie.

### 2.4. Blood Sample Collection and Testing

Blood samples were collected from each participant to measure blood plasma lipid and glucose content. The samples were collected in the human biochemistry laboratory after participants had completed an overnight 12 h fast. Blood was collected from the distal lateral portion of digit II (index finger) using a one-use finger stick needle and a capillary assay tube. The finger stick procedure was completed by a standard approved protocol. The tip of the finger was held under warm water for 10–15 s to promote enhanced blood circulation. The finger was then disinfected with an alcohol wipe, dried with a lint-free cotton pad, and pricked with a finger stick. A total of 40 µg of blood was collected from each participant. The blood was placed into a Lipid Profile Alere Cholestech LDX cassette. Total cholesterol, HDL-C, TG, and blood glucose were measured by a whole blood enzymatic spectrophotometric method, using an Alere Cholestech LDX machine. Plasma LDL-C was estimated using Friedewald equation on the basis that TG > 400 mg/dL.

After each blood sample was collected, the excess blood was absorbed into a separate cotton pad. All used gloves, wipes, and pads were placed into a secure biohazard trash receptacle. Used needles were placed into a closed sharps container for further disposal.

### 2.5. Statistical Analyses

Analyses were performed with the R-studio program for statistical computing and graphics © 2009–2018 RStudio (V.1.1.463), Inc. (Boston, MA, USA). The packages: “naniar” (V. 0.4.2), “caret” (V.6.0-84), “leaps” (V.3.0), “rpart”(V. 4.1-15), “rattle” (V.5.2.0), “rpart.plot”(V. 3.0.8), “RColorBrewer” (V.1.1-2), and “plyr” (V. 1.8.4) were used in the statistical analysis including the creation of the decision trees. NCEP ATP III MetS criteria was used to categorize participants meeting MetS parameters. The data set was split into a training and testing data set with 70% of the data set used for training and 30% for testing. The validity of the models are created with training and evaluated on testing data sets using 10-fold cross-validation.

#### 2.5.1. Decision Trees

Statistical decision trees were created to predict MetS parameters. Missing data for any of the variables resulted in the observation being dropped from the data set. The decision trees were pruned to both prevent statistical overfitting and to allow for the decision tree to provide more practical recommendations to individuals. The average precision, recall, and accuracy were calculated from the 10-fold cross-validation on the testing data set. The accuracy of the model refers to the probability that the decision tree makes a correct prediction. The error of the model refers to the probability that the decision tree makes an incorrect decision. The recall of the model refers to the ability of the decision tree to make a positive diagnosis among individuals who suffer from the symptom. The precision of the model is the likelihood of the decision tree to make a true positive prediction among all positive predictions.

Decisions trees work by branching the data into distinct non-overlapping regions. The tree building process is referred to as ‘top down greedy’ because each successive split which consists of choosing a variable and a node leads to the greatest possible reduction in error. These choices are a result of this statistical procedure, and are chosen by this process, not by the researchers. We input the variables of food group consumption, gender, and exercise for the model to obtain these predictions. This analysis provides a visual and hierarchical representation of the most important inputs for prediction. In this way, the decision trees provide different ways to decrease risk of meeting MetS parameters. Each cut point is associated with a probability that can be used for clinical implications based on the sample data. Decision trees have several advantages: when there is a non-linear or complex relationship between the input variables and the response variable decision trees generally outperform linear regression for the given input variables. Decision trees closely mirror human decision making process, and are easier to explain than linear regression.

#### 2.5.2. Linear Regression Analysis

The multiple regression models are restricted to the male population resulting in 135 observations once data with missing variables were removed. We selected our multiple regression models using the best subsets method considering all variables. We evaluated the models on the testing data set using 10-fold cross-validation on the average mean squared error, proportion of variance explained by the model, and mean absolute error. The best regression models were waist circumference and diastolic blood pressure.

## 3. Results

### 3.1. Demographics and Prevalence of MetS

To analyze the effects of Food Group consumption on MetS parameters, participants were divided by gender. Our sample included 153 males between the ages of 18 and 65 years, with an average age of 23 ± 1.4 years, and 309 females between the ages of 18 and 49 years, with an average age of 25 ± 0.7 years (Table 1).

Approximately 16.7% of the participants met the requirements for MetS [22]. Specifically, 20.9% of the male population, and 14.56% of the female population had MetS. On average, low HDL-C was the most commonly observed MetS parameter, followed by high blood pressure, high waist circumference, high triglycerides, and high blood glucose. The relative prevalence, ranges, and averages of each measured parameter by gender are listed in Table 1. Average parameter differences among those who had MetS and those who did not have MetS are displayed accordingly for both males and females in Table 2.

### 3.2. My Plate Food Group Consumption and Physical Activity

Results stratified by gender of exercise, fruit, vegetable, grain, protein, and empty calorie consumption can be found in Table 3. Protein consumption was the only food group in which participant from both genders met the amount recommended by the MyPlate guidance system. Vegetable consumption was the most deficient among both groups at 25% of recommendation for males and 42% for females. Participants from both genders on average me the recommended exercise week frequency of two or more days/week stablished by the dietary guidelines for Americans.

### 3.3. Metabolic Syndrome Parameters Decision Trees

#### 3.3.1. Blood Pressure

**Figure 1:**
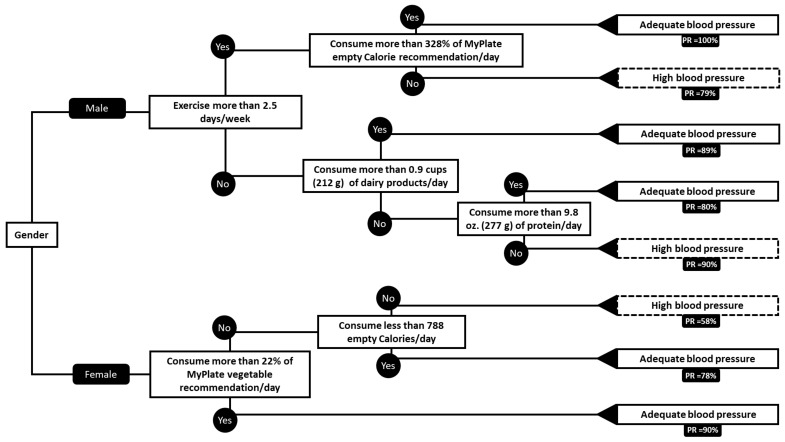
Blood pressure decision tree. All recommendations are based on the MyPlate food guidance system. Blood pressure was assigned as high if above 130/85 mmHg. Abbreviations: PR, prediction rate. The recall rate of the tree is 93.7%. The precision rate is 83.1% and the accuracy rate of the tree is 82.6%.

Approximately 54.3% of the male participants, and 37.9% of the female participants had elevated blood pressure over the MetS indicating threshold of 130/85 mmHg [22] (Table 1). In Figure 1, we created a decision tree to predict whether a participant has high blood pressure or not (Systolic > 130 mmHg; Diastolic > 85 mmHg).

This decision tree begins predicting whether the participant has elevated blood pressure based on gender, dietary factors, and physical activity levels. The variables used to predict elevated blood pressure were gender, exercise, empty calorie percentage (EmptyP), empty calorie amount (EmptyA), grain calorie percentage (GrainP), grain calorie amount (GrainA), vegetable calorie percentage (VegP), vegetable calorie amount (VegA), fruit calorie percentage (FruitA), fruit calorie amount (FruitP), protein calorie percentage (ProtP), protein calorie amount (ProtA), dairy calorie percentage (DairyP), and dairy calorie amount (DairyA) (Supplementary). Data for food group amount, food group percentage, empty-calorie amount, and empty calorie percentage were calculated based on MyPlate recommendations [23].

##### Results in Male Blood Pressure

If the participant is male, predictions for the presence of elevated blood pressure can be viewed on the top half of the decision tree in Figure 1. The first branch addresses the number of days per week the male participants engage in physical activity. The second branch is the effects of dairy consumption and the last branch is the amount of empty calories consumed as weighed by the tree.

For male participants who exercise more than 2.5 times per week, the tree then further directs its decision calculators on the amount of dairy that the participant consumes each day. If the male exercises more than 2.5 times per day and consumes greater than or equal to 0.9 cups (c) (212 g) of dairy each day, the tree predicts that he has a low risk for elevated blood pressure, with a true prediction rate of 89%. If he consumes less than 0.9 c (212 g) of dairy each day, then the tree is concerned with the amount of dietary protein consumed daily. If males exercise more than 2.5 times per day, consume less than 0.9 c (212 g) of dairy each day, and consume more than 9.8 oz (277 g) of protein each day than it is predicted he has a low risk for elevated blood pressure with a true prediction rate of 80%. However, in the same circumstances, our tree predicts that males do have a high risk for elevated blood pressure if they eat less than 9.8 oz (277 g) of protein per day with a true prediction rate of 90%.

For males who exercise less than 2.5 days, the tree queries if the male consumes an empty calorie % ≥ 338%. If the male has levels greater than 338% the tree predicts that he does not have a high risk of elevated blood pressure with a true prediction rate of 100%. If the male has an empty calorie percentage less than 338%, then the tree predicts that he has a high risk of elevated blood pressure, with a true prediction rate of 79%.

##### Results in Female Blood Pressure

If the participant is female, predictions for the presence of elevated blood pressure can be viewed on the bottom half of the decision tree in Figure 1. The tree first queries the percentage recommended vegetable consumption. If consumption is greater than 22% of the vegetable recommendation than the tree predicts that she does not have a high risk for elevated blood pressure, with a true prediction rate of 89%. Alternatively, if vegetable consumption is less than 22% of the amount recommended, then the tree is interested in the total amount of empty calories that she consumes. If vegetable consumption is less than 22% of the recommendation and her empty calorie consumption is less than 788 kcal, then the tree predicts low risk of elevated blood pressure, true prediction rate of 78%. However, if vegetable consumption is less than 22% of recommended, and her empty calories are more than 788 kcal, then the tree predicts high risk for elevated blood pressure, with a true prediction rate of 58%.

In a 10-fold cross-validation, we found that the predictions about blood pressure, made by the decision tree in Figure 1, has a 17.4% error rate. We can interpret this number as meaning that the tree incorrectly predicts if a participant has elevated blood pressure 17.4% of the time. Conversely, this tree had an 82.6% accuracy rate. We can interpret this as the tree correctly predicting if a participant has elevated blood pressure 82.6% of the time. The precision rate of 83.1% means that 83.1% of the prediction of the category ‘adequate blood pressure’ is a correct decision. The advantage of the high blood pressure tree is the high recall rate of 93.7%, which means that the model is correctly able to identify an ‘adequate blood pressure’ individual 93.7% of the time.

#### 3.3.2. HDL-Cholesterol

**Figure 2:**
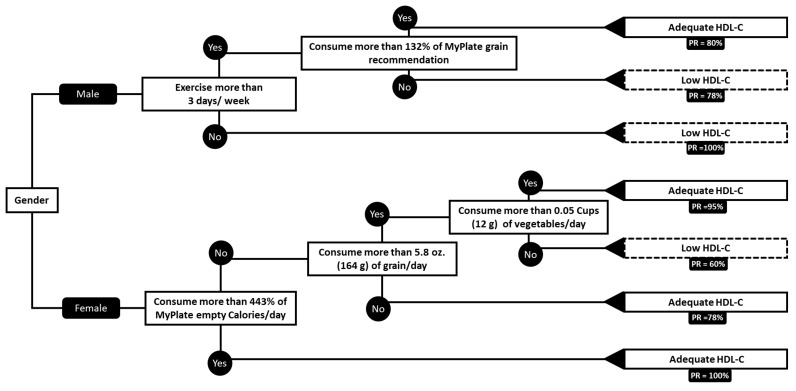
HDL-C decision tree. All recommendations are based on the MyPlate food guidance system. Abbreviations: HDL-C, high-density lipoprotein cholesterol. Low HDL-C was determined based on the NCEP ATP III criteria as circulating levels below 40 mg/dL for males and 50 mg/dL for females. Abbreviations: PR, prediction rate. The recall rate of the tree is 91.8%, the precision rate is 90.3% and the accuracy rate of the tree is 88.0%.

Roughly 50.3% of male participants and 43.4% of females participants had low HDL-C, below the threshold of 40 mg/dL for males, and 50 mg/dL for females [22] (Table 1). In Figure 2, we created a decision tree to predict low HDL-C risk.

The variables used to predict decreased HDL-C were gender, exercise, empty calorie percentage (EmptyP), empty calorie amount (EmptyA), grain calorie percentage (GrainP), grain calorie amount (GrainA), vegetable calorie percentage (VegP), vegetable calorie amount (VegA), fruit calorie percentage (FruitA), fruit calorie amount (FruitP), protein calorie percentage (ProtP), protein calorie amount (ProtA), dairy calorie percentage (DairyP), and dairy calorie amount (DairyA) (Supplementary).

##### Results in Male HDL-C

The results for the predictions of low HDL-C for males can be viewed on the top section of the tree in Figure 2. If a male participant exercises fewer than 3 days per week, the tree predicts that this male has a high risk of low HDL-C with a true prediction rate of 100%.

If males exercise more than 3 days, the tree then focuses on percentage of the recommended amount of grains consumed. If consumption is greater than 132% of the grain recommendation, then the tree predicts that this male does not have high risk of low HDL-C, with a true prediction rate of 80%. If the male gets less than 132% of his grain recommendation, then the tree predicts that this male will have a high risk of low HDL-C with a true prediction rate of 78%.

##### Results in Female HDL-C

For female participants, predictions for the presence of low HDL-C can be viewed on the bottom section of the tree in Figure 2. If a female consumes more than 443% of her recommended empty-calorie percentage, then the tree predicts that the female does not have a high risk of low HDL-C, with a prediction rate of 100%. If the female typically consumes less than 443% then the tree focuses on grain consumption. If grain consumption is less than 5.8 oz (164 g), then the tree predicts that that female will not have a high risk of low HDL-C, with a true prediction rate of 79%. If her grain consumption is greater than 5.8 oz (164 g), then the tree is interested in vegetable consumption. If the vegetable consumption is greater than 0.05 c (12 g), then the tree predicts that the female does not have high risk of having low HDL-C, with a true prediction rate of 95%. If the female does not consume greater than or equal to 0.05 c (12 g) of vegetables, then the tree predicts that she will have high risk for low HDL-C, with a true prediction rate of 60%.

In a 10-fold cross-validation, we found that the predictions regarding HDL-C, made by the decision tree in Figure 2, has an 11.9% error rate. We can interpret this number as meaning that the tree incorrectly predicts a participant’s HDL-C level 11.9% of the time. The advantage of the HDL decision tree is the high accuracy rate of 88.0%. The tree has a high recall rate of 91.8% meaning that the model the model is able to identify an ‘adequate HDL’ level individual 91.8% of the time. In addition, the tree has a high precision rate of 90.3%, meaning that 90.3% of the prediction of the category ‘adequate HDL’ is a correct decision.

### 3.4. Regression Analysis

#### 3.4.1. Waist Circumference in Males

We modeled waist circumference in males. Our best model was constructed using five variables (Diastolic blood pressure (DBP), triglyceride, glucose, exercise, and empty calorie percentage). Our model is of the form:

Predicted Waist Circumference = 15.84 + 0.594 × DBP + 0.041 × Triglyceride + 0.302 × Glucose − 2.096 × Exercise − 0.022 × Empty Calorie Percentage. Table 4 summarizes the waist circumference regression findings.

The model had an adjusted R-squared = 0.5833, meaning our five variable model can explain 58.33% of the variation in male waist circumference. According to our model one unit (mmHg) increase in DBP, ceteris paribus, is associated with a 0.594 cm increase in waist circumference (*p* = 1.31 × 10^−5^). A one-unit increase in triglyceride (mg/dL), ceteris paribus, is associated with a 0.041 cm increase in waist circumference (*p* = 0.000218). A one-unit increase in glucose, ceteris paribus, is associated with a 0.302 cm increase in waist circumference (*p* = 0.0159). One unit increase in the exercise variable, ceteris paribus, is associated with a 2.096 cm decrease in waist circumference (*p* = 0.0166). Note that the exercise variable is defined frequency of exercise where 1 = never, 2 = 1–2 days, 3 = 3–4 days, 4 = 5–6 days, 5 = every day. A one-unit increase in the empty calorie % of goal, ceteris paribus, is associated with a 0.022 cm decrease in waist circumference (*p* = 0.0096).

#### 3.4.2. Diastolic Blood Pressure in Males

We modeled diastolic blood pressure in males. Our best model was constructed using five variables (waist circumference, total cholesterol, vegetable percentage of recommendation, dairy percentage of goal, and protein percentage goal). Our model is of the form:

Predicted DBP = 44.67 + 0.385* (Waist Circumference) + 0.041*(Total Cholesterol) −0.049*(Vegetable % of Goal) − (0.026) × (Dairy % of Goal) + 0.005(Protein% of Goal). Table 5 summarizes the male diastolic blood pressure regression findings.

The model had an adjusted R-squared = 0.5042, meaning our five variable model can explain 50.42% of the variation in male diastolic blood pressure. One cm increase in waist circumference, ceteris paribus, is associated with a 0.385 increase in DBP (*p* = 2.56 × 10^−11^). One unit increase in total cholesterol (mg/dL), ceteris paribus, is associated with a 0.041 increase in DBP (*p* = 0.0181). One unit increase in vegetable % of goal, ceteris paribus, is associated with a 0.049 decrease in DBP (*p* = 0.0254). A one-unit increase in dairy % of goal, ceteris paribus, is associated with a 0.026 decrease in DBP (*p* = 0.0386). A one-unit increase in protein % of goal, ceteris paribus, is associated with a 0.005 increase in DBP (*p* = 0.0214).

### 3.5. Prediction and Modeling of Other MetS Parameters

The remaining MetS parameters were investigated using both decision tree and regression analysis; however, the statistical error rates were higher when cross-validated and it was determined that the BP and HDL-C were the most useful at providing guidance. Prediction of MetS as a binary model was also explored, however, the predictive ability of the model (precision, recall, and accuracy) was lower, and we found that the practical advice from the tree was not intuitive and harder to follow due to the size/depth of the tree.

## 4. Discussion

### 4.1. Decision Trees

We studied the effects of exercise, empty calories, vegetables, fruits, grains, dairy, and protein consumption on MetS parameters. Our model suggests that female blood pressure was most affected by vegetable consumption, empty calorie intake, and/or a combination of the two; while male blood pressure seemed more dependent on exercise, dairy consumption, empty calories, protein intake, and/or a combination of these factors. Next, our model proposes that a female’s HDL-C was most influenced by the number of empty calories, grains, and/or vegetables consumed. Alternatively, exercise, grain consumption, and/or a combination of the two, tended to affect male’s HDL-C levels more than other measured factors.

Differences in lifestyle and dietary patterns may be responsible for the variance of determinants on MetS parameters between male and female populations. For instance, the consumption of vegetables seems to have a major impact on female blood pressure, however, this does not connote that male blood pressure is unaffected by vegetable consumption [24]. Male blood pressure is affected by vegetables, yet, males tend to consume vegetables less frequently than their female counterparts, thus possibly reducing the significance of this dietary variable on their blood pressure [12,25]. Similarly, exercise influences both sexes’ blood pressure. However, studies have suggested that males tend to be more physically active and may even experience a more prolonged decreased-post-exercise-blood-pressure than women [26,27]. This may explain why our decision tree displays exercise as a significant determining factor in male blood pressure, but not in female blood pressure (Figure 1). This does not indicate that blood pressure in females is not affected by exercise [28].

Furthermore, factors affecting HDL-C concentrations also differ between males and females. For example, our decision tree displays that exercise is an important determinant factor of HDL-C status among males but is not represented in the decision tree among the female participants (Figure 1). While physical activity affects HDL-C levels in both males and females, it is reported that women may require a higher volume of physical activity to raise HDL-C levels when compared to their male counterparts [29]. Therefore, physical activity may be a more relevant contributor to male HDL-C levels, which is why it is displayed among males but not females in the decision tree (Figure 2). Nonetheless, our model provides useful indicators of differences and similarities between male and female risk factors of MetS parameters based on participants’ lifestyle choices.

#### 4.1.1. Male Blood Pressure

##### Exercise

Our model suggests that exercise is an important factor in male blood pressure. The tree predicted that males would not have high blood pressure if they participated in physical activity more than 2.5 times per week, in conjunction with either meeting a specific dairy intake or a specific protein intake. Exercise is associated with a decrease in post-activity systolic blood pressure [30]. Specifically, according to the American College of Sports Medicine, blood pressure can be significantly reduced via endurance exercise for up to 22 h after physical activity [31]. Other studies suggest that most types of physical activity can also reduce blood pressure depending on intensity and duration [32]. The study findings are in accordance with the recommended 2 or more days/week of physical activity stated on the 2015 dietary guidelines for Americans [8].

##### Dairy

Males were predicted by our model to have adequate blood pressure if they consumed greater than or equal to 0.9 c (212 g) of dairy each day in combination with exercising at least 2.5 times per week. Several studies have also reported an inverse relationship with dairy consumption and blood pressure [33,34,35]. The calcium content in dairy beverages seems to be the leading contributing factor in systolic and diastolic blood pressure reduction [34]. This association is possibly due to calcium’s influence on blood plasma osmolality [36]. One study even indicated that “lower calcium intake was the most consistent factor in hypertensive individuals” in the United States [35].

##### Protein

Males who participated in physical activity at least 2.5 times per day, consumed lower than 0.9 c (212 g) of dairy, and consumed more than or equal to 9.8 oz (277 g) of protein per day, were predicted to have adequate blood pressure. Our findings were similar to other conducted research about total protein intake and blood pressure [37,38,39,40]. Certain amino acids that are found in protein foods—such as tyrosine, tryptophan, and arginine—may contribute to a reduction in blood pressure by inducing vasodilation [38,39].

##### Empty Calories

Our model shows that males that exercise more than 2.5 days a week and consumed more than 328% of empty-calorie recommendations have low risk of having high blood pressure. Our observation contradicts many studies on the effect of empty calories in blood pressure [10,24]. It is improbable that increase in empty calorie consumption was the underlying factor decreasing the risk of high blood pressure. Although data was collected on exercise frequency, the duration and intensity of the exercise are unknown. Increased exercise duration and intensity are associated with improved blood pressure [29,41,42,43] and it is positively associated with increased overall calorie consumption [44], this may account for the increases in the empty calorie intake.

#### 4.1.2. Female Blood Pressure

##### Vegetables

Women who consumed greater than 22% of their recommended intake, were predicted by our tree to have adequate blood pressure. It is well known that vegetables are typically low in sodium, and rich in potassium. Several studies report that diets high in vegetables, low in sodium, and/or high in potassium protect against hypertension [45,46,47]. There are multiple underlying causes behind this observation such as the pressure regulating effects of extracellular and intracellular concentrations of sodium and potassium [45,46,47]. It is also reported that ascorbic acid found in many vegetables may contribute to decreased systolic and diastolic blood pressure [48]. Ascorbic acid decreases blood pressure, in part, by acting as a diuretic [49,50].

##### Empty Calories

Our tree predicted that women would have high blood pressure if their vegetable consumption was less than 22% and empty calorie consumption was greater than 788 kcal. Low vegetable consumption could contribute to high blood pressure by reasons previously discussed. Additionally, increased empty calorie intake (>788 kcal/day), may have several consequential effects that could increase one’s blood pressure. First, increased consumption of empty calorie saturated fats is linked to an increased risk of plaque formations and atherosclerosis. An increased presence of arterial fatty plaques may decrease and/or inhibit the bioavailability of nitric oxide. Thus, decreasing nitric oxides vasodilating effects on blood vessels, and increasing blood pressure [51,52]. Foods high in empty calories also often contain high sodium and low potassium contents. Literature has reported that elevated sodium and reduced potassium intake is directly related to an increase in systolic blood pressure via pathogenic osmolarity mechanisms [53,54].

#### 4.1.3. Male HDL-Cholesterol

##### Exercise

Our model predicts that men who do not exercise at least 3 times per week will have low HDL-C. However, when they exercise more than 3 days per week and consume a higher amount of grains, they are predicted to have normal HDL-C. Additional studies have also found that exercise is extremely beneficial in raising HDL-C concentrations [55,56]. Dyslipidemia and low HDL-C levels can be improved by a variety of physical activities, depending on their duration, intensity, and type [27,55,56].

##### Grains

Similar to female participants who consumed adequate grains, males who consumed greater than 132% of their recommended GrainP, were predicted by our model to have normal HDL-C. High fiber, low glycemic index, low glycemic load grains may be responsible for this finding [57,58,59].

#### 4.1.4. Female HDL-Cholesterol

##### Empty Calories

We observed in our model that females who typically consume more than 443% of their empty calorie recommendation have a decreased risk for low HDL-C. Although there are multiple empty calorie food sources associated with increased HDL-C such as eggs [60], dairy [61], and unprocessed red meat [62], it is not likely that consumption of those foods is the underlying factor of this observation. Similar to what it was observed on the male branch of the blood pressure decision tree, it is probable that the reason for this observation is the association between high empty calorie intake consumption and increased exercise. It is well established and previously described that one of the most important factors determining HDL-C concentration is physical activity.

##### Grains

Females who typically consume less than 443% of their empty calorie recommendation, and less than 5.8 oz (164 g) of grains per day, were predicted to have normal HDL-C levels according to our decision tree. Similarly, The American Heart Association advocates increasing HDL-C by incorporating a diet that is low in empty calories such as saturated fat, trans fat, and added sugars, while including adequate grain consumption from mostly whole grain and fiber-rich foods [57,58]. When compared to whole grains, non-whole grains typically have a higher glycemic index and glycemic load which can decrease HDL-C [59]. Therefore, lower consumption of total grains, combined with an increase in whole grain foods, can help to increase HDL-C.

##### Vegetables

Our model predicted that females would have normal HDL-C if they consume at least 0.05 c (12 g) Vegetables, in conjunction with getting adequate grains and lower empty calories. A diet high in whole grains and vegetables will be high in soluble fiber. Soluble fiber is known to lower blood lipid levels, and improve total cholesterol to HDL cholesterol levels [63,64], which is consistent with our findings on HDL-C in women.

### 4.2. Regression Analysis

#### 4.2.1. Male Waist Circumference

It has been observed that elevated waist circumference is linked to an increased prevalence of hypertension, hyperglycemia, and dyslipidemia [65]. This could be a plausible reason for why our male waist circumference regression analysis includes these associated metabolic factors. Our model predicates that with an increase in exercise, there will be a decrease in male waist circumference. In contrast, physical inactivity may result in low energy expenditure, which can create an energy imbalance of a caloric surplus [66,67]. This model also indicates increases in empty calories to be associated with decreased WC. Similar to what was observed in the female branch of the HDL-C decision tree, it is likely that this is due to the confounding effects of exercise intensity and duration of the empty calories reported.

#### 4.2.2. Male Diastolic Blood Pressure

Our MLR diastolic blood pressure determinants are in accordance with the BP decision tree (Figure 1) previously shown. The main dietary factors affecting this MetS parameter being: vegetable intake, dairy consumption, and protein intake. The underlying effect of those dietary factors on BP are previously discussed.

## 5. Conclusions

MetS is growing in younger populations. Prevention at early life stages maybe beneficial to decrease the progression of the symptoms. It was observed that many individual parameters have higher rates of prevalence even in this younger population, Table 1.

Our decision tree analysis showed that the MyPlate food guidance system is a useful tool to prevent low HDL-C prevalence and high blood pressure. Nonetheless, food recommendations seem to be more valuable when stratified by gender. This is unlikely to be due to the different effect of food in male and female physiology but rather on the food preferences by gender. Our study provides insight into the importance of tailoring recommendations to food items that are likely to be part of an individual diet rather than foods that are unlikely to be consumed by such individuals. Additionally, this analysis provides practical advice on specific requirements of MyPlate food group consumption to reduce risk of elevated BP, WC, and low HDL-C (Figure 1 and Figure 2). We found that the probability in this data set of male participants having low HDL-C is 100% when they do not exercise more than 3 times a week. Similarly, for females the probability in this data set of having adequate blood pressure is 90% when they consume 22% or more of the MyPlate vegetable recommendation. Finally, our data showed that a significant amount of the variability on WC and DBP in males can be predicted by MyPlate food group recommendation consumption and exercise. It is important to notice that there will always be error on dietary assessments such as a two-day diet record, but it is critical to keep collecting diet information to evaluate specific populations and stablish trends overtime on prevalent issues such as MetS.

## Figures and Tables

**Table 1:** Metabolic syndrome parameter ranges and prevalence by gender

Gender	WC		HDL–C		TG		BP		BG	
	Range (cm)	Prev %	Range (mg/dL)	Prev %	Range (mg/dL)	Prev %	Range (mmHg)	Prev %	Range (mg/dL)	Prev %
Male	62–144	13.1	15–111	50.3	45–577	20.9	71/44–155/106	54.3	69–119	12.4
Female	60–181	19.4	15–149	43.4	45–613	14.2	88/45–150/111	37.9	67–214	7.7

Data show the lower and the higher value recorded (range) and the percentage of participants that met each individual parameter (Prev %) of metabolic syndrome. Abbreviations: WC, waist circumference; HDL-C, high-density lipoprotein cholesterol; TG, Triglycerides; BP, blood pressure; BG, blood glucose; Prev, prevalence.

**Table 2:** Gender general information on metabolic syndrome

Variable	Overall		With MetS		Without MetS	
	M	F	M	F	M	F
Age (years)	23 ± 1.4	25 ± 0.7	24 ± 7.1	30 ± 0	23 ± 1.4	24 ± 0.1
WC (cm)	86.3 ± 14.5	79.8 ± 0.7	100.3 ± 11.3	103.2 ± 12.0	82.6 ± 16.6	76.1 ± 0.1
HDL-C (mg/dL)	41.0 ± 6.3	53.1 ± 2.1	33.2 ± 36.7	41.4 ± 2.8	43.1 ± 6.3	55.1 ± 2.2
TG (mg/dL)	111.6 ± 52.3	104.3 ± 17.6	207.6 ± 241.8	182.1 ± 42.4	85.1 ± 52.3	90.9 ± 17.7
BP (mmHg)	128/81 ± 7.1/8.5	113/79 ± 10.2/2.8	138/89 ± 4.9/6.7	124/89 ± 6.0/12.7	125/79 ± 4.5/8.4	111/77 ± 10.3/2.8
BG (mg/dL)	91.9 ± 2.8	89.0 ± 10.6	96 ± 0	100 ± 33.2	91 ± 2.8	87 ± 10.6

All values are means ± Standard deviations. Abbreviations: MetS, metabolic syndrome; M, male; F, female; WC, waist circumference; HDL-C, high-density lipoprotein cholesterol; TG, triglycerides; BP, blood pressure; BG, blood glucose.

**Table 3:** Physical activity and MyPlate food group consumption by gender

Gender	Fruit		Vegetables		Grain		Protein		Dairy		Empty Calories		Exercise
	Cups	% Goal	Cups	% Goal	Ounces	% Rec	Ounces	% Goal	Ounces	% Goal	Calories	% Goal	Days/Week
Male	1.0 ± 0.1	47	0.8 ± 0.1	25	6.8 ± 0.3	79	11.7 ± 0.5	129	1.9 ± 0.1	64	764.2 ± 89.6	179	3.4 ± 0.09
Female	1.2 ± 0.2	50	1.1 ± 0.1	42	5.6 ± 0.1	77	6.0 ± 0.2	105	1.6 ± 0.1	53	653.2 ± 38.3	223	3.0 ± 0.05

All values are means ± Standard error. % Goal is defined as the percentage of MyPlate food recommendation consumed on that particular food group on average by each gender.

**Table 4:** Waist circumference regression analysis

Variable	Regression Coefficient	Standard Error	*t*-Value	*p*-Value
Constant	15.8	14.8	1.9	0.28
DBP (mm/Hg)	0.6	0.1	4.6	<0.001
TG (mg/dL)	0.04	0.01	3.9	<0.001
Glucose (mg/dL)	0.3	0.12	2.4	0.01
Exercise (days/week)	−2.1	0.8	−2.4	0.02
Empty Calories (%)	−0.02	0.008	−2.6	0.009

Abreviations: DBP, diastolic blood pressure; TG, Triglycerides; BP. Empty calorie % is the percent of empty calories eaten based on the MyPlate recommendation.

**Table 5:** Male diastolic blood pressure circumference regression

Variable	Regression Coefficient	Standard Error	*t*-Value	*p*-Value
Constant	44.7	4.53	9.9	<0.001
WC (cm)	0.4	0.05	7.6	<0.001
TG (mg/dL)	0.04	0.01	2.4	0.02
Vegetables (%)	−0.05	0.02	−2.3	0.02
Dairy (%)	−0.02	0.01	−2.1	0.04
Protein (%)	0.005	0.002	2.3	0.02

Abbreviations: WC, waist circumference; TG, triglycerides. Vegetable, dairy, and protein % is the percent of this food group eaten based on the MyPlate recommendation.

## Supplementary Materials

The following are available online at https://www.mdpi.com/2072-6643/11/12/2892/s1, raw participant data: mets2.csv, HDL-C and BP R-decision tree syntax: Trees.R, male WC, and BP MLR syntax: Regression.R.
